# Supplementary material for: Challenges and Approaches of Culturing the Unculturable Archaea
Source: Biology (Basel). 2023 Dec 7;12(12):1499. doi: 10.3390/biology12121499 (PMC10740628; doi:10.3390/biology12121499)
Supplement: Supplementary file 1 [file biology-12-01499-s001.zip › Table S1.pdf]

Table 1 (Supplementary). Formulation of Media for Halophilic Archaea

Media	Composition	Quantity g/L	Purpose and target archaea<br><br>Examples	References
**High Salt Medium**	Peptone	10	Target archaea include extreme halophilic,<br><br>a) *Halobacterium salinarium* (IAM 12045),<br>b) *Halococcus morrhuae* (IAM 1711)<br>c) *Halobac terium sp*. (Noda),<br>d) *Halobacterium halobium* Rl,<br>e) *Halobacterium cutirubrum*<br>f) *Halobacterium halobium* R1M1,	(Hamana *et al.*, 1985)
	$MgSO_4$-$7H_2O$	2		
	KC1	2		
	$CaCl_2$	2		
	$FeSO_4$-$7H_2O$	0.005		
	$MnCl_2$-$4H_2O$	0.002		
	NaCl.	250		
	pH: 7.2, Temperature: According to experiment, Oxygen: Aerobic condition			
**Medium for Halophilic archaea (DFMZ Medium 1184)**	**Composition**	**g/L**	The target archaea of DFMZ medium are the moderate upper end halophilic archaea	(Atlas, 2004)
	$NaHCO_3$.	0.16		
	$NaCl_2$,	195		
	$MgSO_4.7H_2O$	50.8		
	$MgCl_2.6H_2O$	32.5		
	Yeast extract,	5.0		

	Calcium chloride (KCl)	5.0		
	$CaCl_2.2H_2O$	0.8		
	Sodium bromide (NaBr)	0.6		
	pH 6.7 ± 0.3 at 25ºC			
**Haloarchaea Phosphate solubilisation Medium (HPS)**	**Composition**	**g/L**	Members of Haloarchaea are the<br>• *Haloferax larsenii,*<br>• *Halococcus hamelinensis,*<br>• *Halosarcina* sp,<br>• *Halolamina* sp,<br>• *Haloferax alexandrinus,*<br>• *Natrinema* sp,<br>• *Halobacterium* sp.	(Yadav *et al.*, 2015)
	Glucose	10.0		
	Ammonium chloride NH_4Cl	0.03		
	Yeast extract	1.0		
	$Ca_3(PO_4)_2$ (TCP) or $Ca_{10}(PO_4)_6(OH)_2$ (HA) or rock phosphate	5.0		
	Sodium chloride (NaCl)	195.0		
	Magnesium chloride hexahydrate $MgCl_2.6H_2O$	35.0		
	Magnesium sulfate heptahydrate $MgSO_4.7H_2O$	50.0		
	Potassium chloride KCl	5.0		
	Ammonium sulfate $(NH_4)_2SO_4$	0.5		
	Sodium nitrate $NaNO_3$	1.0		
	Calcium chloride dihydrate $CaCl_2.2H_2O$	0.5		
	Potassium dihydrogen phosphate KH_2PO_4	0.05		
	Traces *Manganese sulfate* heptahydrate $MnSO_4.7H_2O$			
	Traces Ferrous sulfate heptahydrate $FeSO_4.7H_2O$			
	Agar	20		

	pH: 7.4 (adjusted with tris base), After autoclave add 80g/L $NaHCO_3$ and 250g/L sodium pyruvate solutions sterilised by filter should be added to the medium.			
**Media for Dead Sea Halobacteria**	**Composition**	**g/L**	• *Halobacterium sodomense*.	(Oren, 1983)
	Peptone	1		
	Yeast extract	1		
	Potato Starch	20		
	NaCl	29.22		
	$MgCl_2$	~ 122		
	Dead sea water	800ml		
	NaCl.	0.5 M		
	Add distal water to make up the volume 1 litre. (pH 6.5 to 7.0).			
**Basal media**	**Composition**	**g/L**	This is general purpose basal medium. Used for the formulation of different specified media.	(Manikandan *et al.*, 2009)
	$CaCl_2$	1.140		
	$MgCl_2$	5.140		
	$NaHCO_3$	0.200		
	KBr	0.100		
	KCl	0.690		
	$SrCl_2$	0.026	• *Halorubrum sp.* VKMM 017	
	Na_2SO_4	4.060	• *H. salinarum* VKMM 013,	
	NaF	0.003	• *Halogeometricum borinquense* VKMM 001	
	$NaSiO_3$	0.002		
	NaCl	24.320		

	$FeSO_4$	0.001	• *Haloferax sp*. VKMM 026,	
	H_3BO_3	0.027		
	Nutrient broth	13		
	Prepared up to one Litre in distilled water.			
**Marine salt (S.W.) containing Basal media**	Basal media of marine salts (S.W.) contain 3.4 M sodium chloride (NaCl).		• *Haloferax mediterranei* Sech7a<br>• *Haloarchaeon* Sech7a.<br>• *Haloarcula sinaiiensis ATCC 33800,*<br>• *Haloarcula marismortui DSM 3752,*<br>• *Haloarcula hispanica DSM 4426,*<br>• *Haloarcula californiae ATCC 33799,*<br>• *Haloarcula japonica DSM 6131,*<br>• *Haloarcula vallismortis DSM 3756,*<br>• *Halobacterium halobium CCM 2090,*<br>• *Halobacterium halobium CECT 396,*<br>• *Halobacterium salinarum CCM 2148,*<br>• *Halobacterium salinarum DSM 3754,*	(Manikandan *et al.*, 2009)
	Agar	20		
	Yeast extract	5		
	pH 7.5,<br>In case of broth no agar is added, and growth is measured in term of turbidity at 600 nm, Spectro.			
**Eimhjellen medium**	Yeast extract:	15		(Lizama *et al.*, 2001)
	Magnesium sulfate heptahydrate ($MgSO_4$ 7 H_2O)	12		
	$CaCl_2$ 2 H_2O	5		
	NaCl	250		
	Yeast extract	10		

	**Sehgal and Gibbons medium:**	Casamino acids	7.5		(Sehgal and Gibbons, 1960)
		Na citrate	3		
		MgSO4 7 H_2O	12.0		
		KCl	2		
		$FeCl_2$	0.023		
		NaCl	250		
	**M.H. medium**	Proteose-peptone	5		(Torreblanca *et al.*, 1986)
		Yeast extract,	10		
		Glucose	1		
		Penicillin	500 units/ml		
	**HE medium (Hay extract media)**	Yeast extract	0.5		(Torreblanca *et al.*, 1986)
		glucose	0.1		
		25% (w/v) of total salts. The stock of total salts at 30% was prepared as described :			
		NaCl	23.4		
		$MgCl_2$ 6 H_2O	4.2		
		$MgSO_4$ 7 H_2O	6.0		
		$CaCl_2$ 2 H_2O	0.1		
		KCl	0.6		
		$NaCO_3H$	0.02		
		NaBr	0.07		
		$FeCl_3$	0.0005		
		Distilled water	100ml		

	Agar	20		
	Penicillin	500 units/ml		
	pH 7.3 with 1 N KOH before autoclaving.			
***Halobacterium Salinarum* (H.S.) Medium**	NaCl	250	• *Halobacterium salinarum,*<br>• *H. salinarum* strain R1.	(HASAN and MOHAMMADIAN, 2011)
	KCl	2		
	$MgSO_4$	20		
	Tri-Na-Citrate	3		
	$FeSO_4.7H_20$	0.02		
	Casamino acids	5		
	$MnCl_2.4H_20$	0.026		
	Yeast extract	5		
	Agar	20		
	pH: 7			
**Mineral salts medium**	NaCl	2	• *Haloferax* sp. D1227,<br>• *Haloferax volcanii* DSMZ 3757,<br>• *Halococcus morrhuae* DSMZ 1307,<br>• *Halobacterium salinarum* DSMZ 668,<br>• *Haloarcula marismortu*i DSMZ 3752.	(Mevarech and Werczberger, 1985; Cuadros-Orellana *et al.*, 2006)
	KCl	3.75		
	NH_4Cl	0.267		
	K_2HPO_4	0.174		
	$MgSO_4$	37		
	$CaCl_2$	0.5		
	Trace Elements	0.1% (v/v)		
	pH: 7.2			
	Vitamin solution 0.1% (v/v) after autoclave through syringe filter.			
	**Additional supplementation**:			

	Mineral salts solution with			
	yeast extract	0.3%		
	tryptone	0.3%		
	Agar (if required)	20		
	Temperature	40 °C,		
	p-hydroxybenzoic acid:	0.4 mM.		
