# Supplementary material for: Challenges and Approaches of Culturing the Unculturable Archaea
Source: Biology (Basel). 2023 Dec 7;12(12):1499. doi: 10.3390/biology12121499 (PMC10740628; doi:10.3390/biology12121499)
Supplement: Supplementary file 1 [file biology-12-01499-s001.zip › Table S2.pdf]

Table 2 (Supplementary). Formulation of Media for Methanogenic Archaea

Media	Composition	Quantity g/L	Examples	References
**Modified LPBM media also called Acido-Thermophile media**	KH_2PO_4	0.75	*Methanosarcina mazeii* TMA	(Asakawa *et al.*, 1995)
	K_2HPO_4	0.75		
	NH_4CI	1.0		
	$MgC1_2.6H_20$	0.36		
	Sodium acetate	2.5		
	Yeast extract	0.1		
	Polypepton	0.1		
	Trimethylamine hydrochloride	8.0		
	Trace mineral solution	9ml		
	Vitamin mixture	10ml		
	Resazurin solution (0.2%)	0.5ml		
	L-cysteine hydrochloride.$9H_20$	0.5		
	$Na_2S.\ 9H_2\,O$	0.5		
	$NaHCO_3$	0.8		
	Adjust the final pH at 6.8,<br><br>1.7% agar is required for solid media preparation			
	**Composition**	***Quantity***		

**SAB-Medium**	Glucose (20 mM)	*0.0036*	• *Methanobrevibacter smithii,*<br>• *Methanobrevibacter oralis,*<br>• *Methanosphaera stadtmanae,*<br>• *Methanomassilicoccus luminyensis* and<br>• *Methanobrevibacter arboriphilicus* have been cultured from human digestive microbiota.<br><br>Similarly, two mesophile methanogens<br><br>• *Methanobacterium beijingense and*<br>• *Methanosaeta concilii*	(Khelaifia et al., 2016; Atlas, 2004)<br><br>(Balch et al., 1979; Khelaifia et al., 2013)
	$NiCl_2 . 6H_2O$	1.5 mg/L		
	$FeSO_4 . H_2O$,	0.5 mg/L		
	$MgSO_4 . 7H_2O$	0.8 g/L		
	KH_2PO_4	0.5 g/L		
	K_2HPO_4	0.5 g/L		
	KCl	0.05 g/L		
	$CaCl_2 . 7H_2O$	0.05 g/L		
	NaCl	*1.5* g/L;		
	NH_4Cl	1 g/L		
	$MnSO_4 . 7H_2O$	0.6 mg/L;		
	$ZnSO_4 . 7H_2O$	0.1 mg/L;		
	$CuSO_4 . 5H_2O$	0.02 mg/L;		
	$KAl(SO_4)_2 . 12H_2O$	0.2 µg/L;		
	H_3BO_3,	7 µg/L		
	$CoSO_4 . 7H_2O$,	4 µg/L;		
	$Na_2MoO_4 . 2H_2O$	0.5 mg/L;		
	$Na_2SeO_3 . 5H_2O$	3 µg/L;		
	$Na_2WO_4 \times 2H_2O$	4 µg/L;		
	Nitrilotriacetic acid,	15 mg/L;		
	Sodium acetate	1 g/L;		

	Trypticase	2 g/L;		
	Yeast extract	2 g/L		
	L-cysteine hydrochloride monohydrate	0.5 g/L		
	Valeric acid	5 mM;		
	Isovaleric acid	5 mM;		
	2-methylbutyric acid	5 mM;		
	Isobutyric acid,	6 mM;		
	2-methyl valeric acid	5 mM;		
	Resazurin,	1 mg/L.		
	Pressure	2.5 bar		
**Aerobic culture with antioxidants**	Prepared SAB media (broth)	5 ml	*M. smithii and B. thetaiotaomicron.*	(Khelaifia *et al.*, 2013, 2016)
	Ascorbic acid	1		
	Uric acid	0.1		
	Glutathione	0.1		
	pH 7.5 (adjust with the help of KOH.			
**Enrichment Media**	**Composition**	**quantity**	This media is used for the isolation of methanogen archaea from a number of sources including water, wastewater, gastro-intestinal tract etc, and be equally applied for the	(Balch *et al.*, 1979; Whitman *et al.*, 2006),
	Yeast extract	2.0 g/L		
	Trypticase peptones	2.0 g/L		
	Salt solution A*	10 mL/L		
	K_2HPO_4 –$3H_2O$	0.4 g/L		

	Resazurin solution	0.001 g/L	isolation of archaea from other different sources.	(Wolin *et al.*, 1963), (Bryant *et al.*, 1971)
	Sodium acetate	1.36 g/L		
	$NaHCO_3$	5.0 g/L,		
	Cysteine/hydrochloride	0.5 g/\L,		
	Sulfide solution	20 mL/L.		
	Trace element solution	10 mL,		
	Vitamin solution	10 mL,		
	***Preparation of Salt solution A**			
	NH_4Cl	100 g/L,		
	$MgCl_2.6H_2O$	100 g/L,		
	$CaCl_2.2H_2O$	40 g/L.		
	Dissolve in 800 ml distilled water, adjust the pH to 4 by HCl.			
	**Trace element solution:**			
	Nitrilotriacetic acid	1.5 g/L,		
	$Fe(NH_4)_2(SO_4)_2.6H_2O$	0.2 g/L		
	Na_2SeO_3	0.2 g/L,		
	$CoCl_2 .6H_2O$	0.1 g/L,		
	$MnSO_4.2H_2O$	0.1 g/L,		
	$Na_2MoO_4.2H_2O$	0.1 g/L,		
	$Na_2WO_4.2H_2O$	0.1 g/L,		
	$ZnSO_4 .7H_2O$	0.1 g/L,		

	$AlCl_3.6H_2O$	0.04 g/L,		
	$NiCl_2.6H_2O$	0.025 g/L,		
	H_3BO_3	0.01 g/L,		
	$CuSO_4 .5H_2O$	0.01 g/L.		
	Dissolve all these chemicals in 800 ml, adjust the pH to 6.5 with KOH and make the volume up to 1000ml.			
	**Vitamin solution:**			
	Ca pantothenate	10 mg/L		
	Pyridoxine hydrochloride	10 mg/L,		
	Riboflavin	10 mg/L,		
	Thiamine hydrochloride	10 mg/L,		
	Biotin	5 mg/L		
	Folic acid	5 mg/L		
	a-Lipoic acid	5 mg/L		
	Vitamin B_{12} (dark stored at 50 ^{o}C)	5 mg/L		
	p-Aminobenzoic acid	10 mg/L		
