# Supplementary material for: Challenges and Approaches of Culturing the Unculturable Archaea
Source: Biology (Basel). 2023 Dec 7;12(12):1499. doi: 10.3390/biology12121499 (PMC10740628; doi:10.3390/biology12121499)
Supplement: Supplementary file 1 [file biology-12-01499-s001.zip › Table S3.pdf]

Table 3 (Supplementary). Formulation of media for Thermophilic and Hyper-thermophilic Archaea

Media	Composition	Quantity g/L	Examples	References
**Hyper thermophilic Archaebacteria**	Artificial sea water is prepared by Kester et al. 1967 protocol with slightly modifications.		*Pyrococcus furiosus*	(Kester *et al.*, 1967; Brown and Kelly, 1989)
	**Solution A:**			
	Sodium chloride	47.8 g/L		
	Sodium sulfate	8 g/L		
	Potassium chloride	1.4 g/L		
	Sodium bicarbonate	0.4g/L		
	Potassium bromide	0.2 g/L		
	Boric acid	0.06 g/L		
	**Solution B:**			
	Magnesium chloride	21.6g/L		
	Calcium chloride (dehydrated)	3 g/L		
	Strontium chloride.(H_2O)	0.05 g/L		
	**Solution C:**			
	Amonium chloride	12.5 g/L		
	Dipotassium phosphate	7.0 g/L		
	Sodium acetate	50.0 g/L		

	Equal volume of solution A and B (440 ml each) mixed and add Yeast extract 1g/L and Tryptophane 5 g/L, dissolve by stirrer and autoclaved. 20 ml of solution should be added separately sterilised through syringe filter.			
**Culture of Thermophilic Enrichment Media. (The medium for the enrichment of methanogens)**	The media is prepared in Hungate tube or 50 ml vial as described by Balch et al., 1979)		*Thermococcales*	(Harmsen *et al.*, 1997)
	Sea Salts,	30 g/L;		
	Piperazine N, N9-bis-ethanesulfonic acid (PIPES) buffer, 3.46 gPIPES,	3.46 g PIPES,		
	NH4Cl,	1 g/L,		
	Sodium acetate,	1 g/L,		
	$NaHCO_3$	1 g		
	KH_2PO_4	0.35 g/L,		
	Yeast extract	2 g/L,		
	Resazurin	1 mg/L.		
	Peptone	2 g/L,		
	Selenite tungstate solution	1 ml,		
	Trace element mixture	1 ml,		
	Vitamin B12 solution	1 ml,		
	Vitamin mixture	1 ml,		
	Growth-stimulating factors (GSF),	1 ml,		

	Thiamine solution,	1 ml,		
	pH 6.5<br><br>H2-CO2 is used in gas phase.			
**Heterotrophic sulfur reducers were enriched in a medium**	The media is prepared in Hungate tube or 50 ml vial as described by Balch et al., (1979)			(Harmsen *et al.*, 1997)
	Sea Salts	30 g/L,		
	NH_4Cl	1 g/L,		
	KH_2PO_4	0.35 g/L,		
	PIPES buffer	3.46 g/L;		
	Yeast extract	1 g/L,		
	Peptone	2 g/L,		
	Sulfur	10 g/L,		
	Mineral solution (Balch et al., 1979)	10 ml		
	Vitamin solution (Balch et al., 1979)	10 ml		
	Resazurin	1 mg.		
	pH 7<br><br>N2 is used in gas phase			
**Medium for the enrichment of**	The media is prepared in Hungate tube or 50 ml vial as described by Balch et al., (1979)			(Balch *et al.*, 1979)
	Sea Salts	30 g/L,		

**autotrophic sulfur reducers**	NH_4Cl	1 g/L,	*Archaeoglobus, Thermocladium* and *Caldivirga*	
	KH_2PO_4	0.35 g/L,		
	MES [2-(N-morpholino) ethanesulfonic acid] buffer	1.95 g/L,		
	$NaHCO_3$	1 g/L,		
	Sulfur	10 g/L,		
	Resazurin	1 mg/L		
	Trace element mixture	1 ml,		
	Selenite-tungstate solution	1 ml,		
	Vitamin mixture (Widal et al., 1992)	1 ml,		
	Thiamine solution	1 ml,		
	Vitamin B12 solution	1 ml,		
	Growth-stimulating factors	1 ml,		
**Basal Mineral Medium**	***Macro-elements**:*		*Pyrobaculum gen.* Hyperthermophilic Neutrophilic	(Allen, 1959; Huber *et al.*, 1987)
	Ammonium sulphate	0.01 M,		
	potassium dihydrogenposphate	0.002 M,		
	Magnesium sulphate	0.001 M,		
	Calcium chloride	0.0005 M,		
	Hydrogen thioperoxide	~ 0.001 M,		
	Micro-element:			

	Manganese	0.5 mg/L,		
	Boran	0.5 mg/L,		
	Iron	4 mg/L,		
	Zinc	0.05 mg./L,		
	Copper	0.02 mg/L,		
	Molybdenum	0.01 mg/L,		
	Vanadium	0.01 mg/L,		
	Sodium sulfide	0.05%		
	Resazurin	0.0001%.		
	Yeas extract	0.02%		
	Peptone.	2.0%		
**Sylvan Spring's medium**	**Basal salt media for Sylvan Spring's Medium (compositions are per litre in water)**		*Thermogladius shockii*	(Meyer-Dombard *et al.*, 2012)
	$NaHCO_3$	0.36 g,		
	$MgCl_26H_2O$	1.67 mg		
	Na_2SO_4	0.27 g,		
	NaCl	0.5 g		
	Yeast	3 g		
	KCl	0.04 g		

**Trace element solution recipes**	All media were a yeast-sulfur (Y.S.) formulation, and received (per L)			
**Medium SS1**	$SrCl_{2}.6H2O$.	0.06 mg		(Wolin *et al.*, 1963)
	RbCl,	0.36 mg		
	$PbCrO_{4}$,	2.0 lμg		
	$CdSO_{4}$.	0.5 lg		
	$ZnSO_{4}.7H_{2}O$,	0.1 mg		
	$VOSO_{4}3.5H_{2}O$,	4.0 lg		
	$Na_{2}MoO_{4}.2H_{2}O$,	0.16 mg		
	$H_{2}WO_{4}$,	0.12 mg		
	$CuSO_{4}.5H_{2}O$,	0.8 lg		
	$CoCl_{2}.6H_{2}O$,	0.1 lg		
	$CaCl_{2}$ $2H_{2}O$,	15.77 mg		
	$BaCl_{2}.2H_{2}O$,	0.07 mg		
	$MnCl_{2}4H_{2}O$,	0.09 mg		
	$AlK(SO_{4})212H_{2}O$,	6.89 mg		
	$FeSO_{4}7H_{2}O$,	0.06 mg		
	yeast extract (Difco),	3 g		
	10 mL N/P solution (containing per liter of water:			
	$NH_{4}Cl$, 444.8 mg			

	$NaNO_3$, 34.2 mg		
	K_2HPO_4) 83.0 mg		
	2% resazurin solution (redox indicator), 0.5 mL		
	MES (2-[N-morpholino] ethane sulfonic acid) as a pH buffer. 3 g		
**Medium WTE**	Medium WTE was obtained by adding the following to 1 L of the 'Sylvan Spring' basal salt solution:		
	$CaCl_2.2H_2O$, 0.5 mg		
	$FeSO_4.7H_2O$, 0.5 mg		
	$AlK(SO_4)_2.12H_2O$, 0.05 mg		
	$MgCl_2.6H_2O$, 15 mg		
	$MnSO_4$, 0.5 g		
	$CoCl_2.$ $6H_2O$, 0.5 mg		
	$CuSO_4.12H_2O$, 0.05 mg		
	$Na_2MoO_4.2H_2O$, 0.05 mg		
	$ZnSO_4.7H_2O$, 0.5 mg		
	H_3BO_3, 0.05 mg		
	nitriloacetic acid and 7.5 mg		
	NaCl (Wolin et al. 1963). 5 mg		
**Medium WTE200**	Medium WTE200 is prepared by concentrating the WTE recipe 200-fold		(Grant and Pramer, 1962)
