# Supplementary material for: Challenges and Approaches of Culturing the Unculturable Archaea
Source: Biology (Basel). 2023 Dec 7;12(12):1499. doi: 10.3390/biology12121499 (PMC10740628; doi:10.3390/biology12121499)
Supplement: Supplementary file 1 [file biology-12-01499-s001.zip › Table S4.pdf]

Table 4 (Supplementary). Formulation of Media for Mesophilic Archaea

Media	Composition	Quantity	Examples	References
**Basal Salts Medium**	The basic media for mesophilic include		Soil Crenarchaeotes	(Repaske, 1956), (Simon *et al.*, 2005)
	KH_2PO_4,	5 mM		
	Na_2HPO_4,	5 mM		
	$(NH_4)SO_4$,	1 mM		
	KCl,	2 mM		
	Vitamin solution ,	1%		
	Daniels' mineral elixir	1%		
	pH 7.0			
	$NaHSO_3$,	2.5 mM		
	Carbenicillin and streptomycin,	100 mg/liter		
	Rifampin (in methanol),	200 mg/liter		
	Cephalothin	6.7 mg/liter		
	Clindamycin	1.7 mg/liter		
	0.2× root extract (1× root extract is 25 g [wet root weight]/liter H_2O),			
	The inhibitors are used to inhibit the growth of bacteria. They include antibiotic and enzymes (lysozyme).			
