# Supplementary material for: Challenges and Approaches of Culturing the Unculturable Archaea
Source: Biology (Basel). 2023 Dec 7;12(12):1499. doi: 10.3390/biology12121499 (PMC10740628; doi:10.3390/biology12121499)
Supplement: Supplementary file 1 [file biology-12-01499-s001.zip › Table S5.pdf]

Table 5 (Supplementary). Formulation of Media for Acidophiles Archaea

Media	Composition	Quantity	Examples	References
**Brock Medium**	The composition of media (per litre in distilled water)		*Sulfolobus*	(Brock *et al.*, 1972)
	$(NH_4)_2SO_4$, ,	1.3g		
	KH_2PO_4,	2.80g,		
	$MgSO_4 \cdot 7H_2O$,	2.50g,		
	$CaCl_2 \cdot 2H_2O$,	0.70g,		
	$MnCI_2.4H_2O$,	1.8mg,		
	$Na_2B_4O_7.10H_2O$	4.5mg,		
	$ZnSO_4.7H_2O$	0.22 mg,		
	$CuC1_2$. $2.H_20$,	0.05mg,		
	$Na_2MoO_4 \cdot 2H_2O$,	0.03 mg,		
	$VOSO_4.2H_2O$,	0.03 mg,		
	$CuSO_4$,	0.01 mg,		
	pH 2-3 (adjusted by 10 N H_2SO_4).			
**M88**	The M88 media is selective for the culturing of thermophilic and acidophilic archaea. The medium is recommended by DSMZ. The M88 is modified form of Brick's medium.		*Thermoacidophilic Acidianus*	(Giaveno *et al.*, 2013)

	• Brock media is added with 10g/L yeast extract, and adjust the pH 2 by 10 N H2SO4<br>• 5.0 g/L Sulphur is added		
**M88+sulphur (M88+S)**	The M88+S media is basically M88 media which is supplemented only with Sulphur.		
**Solid Media**	In solid media for the isolation of archaea the solidifying agent used is Gellan Gum The composition of the media includes solution A, solution B and solution C.		

**Solution A:**<br><br>Gelrite (0.8%) 16 g/L (Dense Layer)	16 g/L
**Solution B:**<br><br>Gelrite (0.4%) 8 g/L (Light layer)	8 g/L
**Solution C:**<br><br>Solution C is composed of **OK** media aided with	
NH4)2SO4	6.0,
MgCl2·8 mM	0.76
Cl	0.2
K2HPO4	1.0

	$MgSO4·7H2O$ — 1.0<br>Casamino acids 2 % — 2.0<br>NaCl 2 mM — 0.12<br>$K2S4O6·20$ mM — 6.05<br>$Ca(NO3)2·4H2O$ — 0.028<br>pH 2.0<br><br>The double layer has positive impact on the growth of thermophilic archaea.		
**Mineralisation Medium**	Mineralisation medium is modified Brock's medium.<br><br>Modifications are<br>pH 3.5<br>Yeast extract: 0.1% — 0.1%<br>D-saccharose: 0.2% — 0.2%<br>Agitation 170 rpm<br>These modifications are followed by addition of the following ingredients convert into mineralisation media.<br>$FeSO_4$: 10mM — 10mM<br>NaH_2PO_4: 10mM — 10mM<br>Adjust pH 4.5.	*Sulfolobus acidocaldarius*	(Miot *et al.*, 2017)

**Basal Medium**	It's Brock medium with pH 3.0 adjusted by H_2SO_4. The component of media includes all ingredients as Brock media except lacking the Carbon source (Brock *et al* (1972).		• S. *acidocaldarius* DSM 639,<br>• *S. solfataricus* P2,<br>• *S. islandicus* Rey15A,<br>• *S. tokodaii* str. 7 and<br>• *Sulfolobus shibatae* B12	(Quehenberger *et al.*, 2019)
